# African Malaria Control Programs Deliver ITNs and Achieve What the Clinical Trials Predicted

**DOI:** 10.1371/journal.pmed.1001088

**Published:** 2011-09-06

**Authors:** Thomas P. Eisele, Richard W. Steketee

**Affiliations:** 1 Department of Global Health Systems and Development, Tulane University School of Public Health and Tropical Medicine, New Orleans, Louisiana, United States of America; 2 Malaria Control and Evaluation Partnership in Africa (MACEPA)-PATH, Seattle, Washington, United States of America

## Abstract

Thomas Eisele and Richard Steketee discuss new research in *PLoS Medicine* by Stephen Lim and colleagues that examined the association of insecticide-treated nets with the reduction of *P. falciparum* prevalence in children under 5 and all-cause post-neonatal mortality.

**Linked Research Article**

This Perspective discusses the following new study published in *PLoS Medicine*:

Lim SS, Fullman N, Stokes A, Ravishankar N, Masiye F, et al. (2011) Net Benefits: A Multi-Country Analysis of Observational Data Examining Associations between Insecticide-Treated Mosquito Nets and Health Outcomes. PLoS Med 8(9): e1001091. doi:10.1371/journal.pmed.1001091

Stephen Lim and colleagues report findings from a multi-country analysis of household survey data on the association between possession of insecticide-treated mosquito nets and child mortality and parasitemia. Scale-up of net coverage was associated with a substantial reduction in childhood mortality and in parasitemia prevalence.

## New Evidence for ITN Effectiveness

There is robust evidence of the efficacy of insecticide-treated mosquito nets (ITNs) in reducing malaria parasite prevalence, incidence, and all-cause child mortality from carefully conducted trials in sub-Saharan Africa across a range of transmission settings [1]. Trials have shown ITNs to both significantly reduce *Plasmodium falciparum* prevalence among children under 5 years old by 13% and post-neonatal (1–59 months) all-cause mortality by 18% in areas of stable malaria transmission in Africa [1],[2]. However, there have been limited data on the effectiveness of ITNs under routine program conditions at preventing malaria morbidity and child mortality, especially at the national level. This has of course raised serious concerns abou how likely the efficacy of ITNs from trials is translating into real-world effectiveness on the ground. There are certainly examples where a proven effective intervention achieved disappointing results when programs ran into constraints with deployment at wide-scale implementation [3],[4].

Stephen Lim and colleagues, in an article published in this week's *PLoS Medicine*, should be commended for their rigorous and systematic analysis of national cross-sectional survey datasets in sub-Saharan Africa assessing the association of ITNs on reducing *P. falciparum* prevalence in children under 5 and all-cause post-neonatal mortality, while controlling for contextual and potential confounding factors [5]. The results show ITN household possession to be associated with a 20% significant reduction in *P. falciparum* prevalence (from seven surveys in seven countries) and a 23% significant reduction in all-cause child mortality (from 29 surveys in 22 countries). Importantly, these results were consistent across a range of malaria transmission settings and across countries with disparate levels of ITN household coverage. They are also consistent with data from smaller-scale studies that have shown ITNs to be associated with significant reductions in malaria under program conditions [6]–[10].

The ITN represents a brilliant intervention—it provides individual protection to the person sleeping under it from infected mosquitoes; the insecticide kills mosquitoes that seek a blood meal thereby reducing the overall propensity for transmission in the community [11],[12]; and if the person under the net is already infected with the malaria parasite, the ITN prevents them from infecting mosquitoes and leading to more transmission. The ITN is tailored to the biology of the African malaria-carrying *Anopheles* mosquitoes that prefer to bite humans, bite late at night when people are sleeping (hopefully under an ITN), and rest on vertical surfaces (such as the walls of the ITN) while they digest their blood meal. National ITN mass distribution campaigns have achieved remarkably high household coverage, even among the most poor and rural areas [13]–[15]. Despite unsubstantiated anecdotes of misuse and non-use [16], given sufficient access to ITNs people use them for protection against malaria [17].

Still, ITNs are not the sole answer to malaria control, and they cost money and need to be continually replaced when they wear out.

## Relevance to Malaria Control

Funding for malaria control has increased dramatically from ∼US$100 million available in 2003 to ∼U$1.5 billion available in 2010, with over three-quarters going to sub-Saharan Africa [18]. Largely based on the results of the ITN trials, there has been a considerable ”leap of faith” by international donors and ministries of health across Africa in relying on ITNs as a cornerstone malaria prevention tool that will translate into real gains on the ground in reducing the malaria burden. To this end between 2004 and 2010, manufacturers delivered more than 400 million nets, with 290 million delivered since 2008, which is sufficient to cover nearly 80% of populations at risk of malaria in Africa [18].

After nearly a decade of investment in malaria control across Africa, there has been a critical need to evaluate the impact this effort has had on reducing the malaria burden, especially for child mortality. However, because nearly all national programs are scaling-up to achieve fullcoverage of populations at risk of malaria, evaluators must rely on an ecological, or plausibility, study design whe attempting to assess the impact of malaria control investments [19]–[21], whereby changes in intervention coverage are measured against simultaneous changes in malaria morbidity and mortality. If the malaria burden is observed to decrease concurrently with intervention coverage in the population, then one deems it plausible tha the program contributed to the improved outcomes observed. This is especially true for ITNs where there are robust empirical data from trials on their efficacy. However, the study design is strengthened immensely when there is additional evidence that the effect seen in trial translates to effectiveness on the ground.

Lim and colleagues have provided timely and vital validating evidence that national programs can decrease malaria morbidity and child mortality through program investments in ITNs. In a world of shrinking resources for global health programs, such evidence is critically important. To emphasize this point, consider what it would have meant if the analysis by Lim and colleagues had shown that despite the evidence from trials, ITNs have no demonstrable association with reducing malaria morbidity and child mortality under program conditions in Africa; this would have been devastating to the integrity of past and future investments in ITNs as a primary tool in the fight against malaria. In fact, they found the opposite and confidence, renewed attention, and investment should follow.

## Next Steps

The next 5 to 10 years will be critical in the fight against malaria, especially if elimination in areas of Africa is to be achieved. As Lim and colleagues suggest, continued scale-up of long-lasting ITNs (LLINs) must be a cornerstone of this effort and there are still lives to be saved with this intervention. LLINs typically wear out after 2–3 years and thus the malaria control community must attend to finding the most efficient means of replacing worn out nets once high coverage has been achieved [22]. And, ITNs alone are insufficient to completely eliminate malaria transmission in areas of Africa suitable to perennial transmission [23]. It is therefore imperative for the malaria community to apply its program experience and success with ITNs towards a focus on testing new tools and delivery approaches to achieve the next level of malaria transmission reduction beyond what is achievable by high ITN coverage alone [24]–[26].

## Abbreviations

ITN: insecticide-treated mosquito net
